# A multi-modal data resource for investigating topographic heterogeneity in patient-derived xenograft tumors

**DOI:** 10.1038/s41597-019-0225-0

**Published:** 2019-10-31

**Authors:** Satwik Rajaram, Maike A. Roth, Julia Malato, Scott VandenBerg, Byron Hann, Chloe E. Atreya, Steven J. Altschuler, Lani F. Wu

**Affiliations:** 1 Department of Pharmaceutical Chemistry, University of California San Francisco, San Francisco, California USA; 2 Lyda Hill Department of Bioinformatics and Department of Pathology, University of Texas Southwestern Medical Center, Dallas, Texas USA; 3 Helen Diller Family Comprehensive Cancer Center, University of California San Francisco, San Francisco, CA USA; 4 Department of Pathology, University of California San Francisco, San Francisco, CA USA; 5 Biorepository and Tissue Biomarker Technology Core, Helen Diller Family Comprehensive Cancer Center, University of California San Francisco, San Francisco, CA USA; 6 Division of Hematology/Oncology, Department of Medicine, University of California San Francisco, San Francisco, CA USA

**Keywords:** Tumour heterogeneity, Multicellular systems

## Abstract

Patient-derived xenografts (PDXs) are an essential pre-clinical resource for investigating tumor biology. However, cellular heterogeneity within and across PDX tumors can strongly impact the interpretation of PDX studies. Here, we generated a multi-modal, large-scale dataset to investigate PDX heterogeneity in metastatic colorectal cancer (CRC) across tumor models, spatial scales and genomic, transcriptomic, proteomic and imaging assay modalities. To showcase this dataset, we present analysis to assess sources of PDX variation, including anatomical orientation within the implanted tumor, mouse contribution, and differences between replicate PDX tumors. A unique aspect of our dataset is deep characterization of intra-tumor heterogeneity *via* immunofluorescence imaging, which enables investigation of variation across multiple spatial scales, from subcellular to whole tumor levels. Our study provides a benchmark data resource to investigate PDX models of metastatic CRC and serves as a template for future, quantitative investigations of spatial heterogeneity within and across PDX tumor models.

## Abstract (summary)

**Table:** 

Measurement(s)	transcription profiling assay • gene mutation • protein profiling assay • neoplastic cell • hematoxylin and eosin stain
Technology Type(s)	RNA sequencing • DNA sequencing • RPPA protein profiling assay • immunofluorescence microscopy assay • brightfield microscopy
Sample Characteristic - Organism	Homo sapiens/Mus musculus xenograft

Machine-accessible metadata file describing the reported data: 10.6084/m9.figshare.9892316

## Background & Summary

Heterogeneity is a key challenge for diagnosing and treating cancer. Solid tumors can contain a vast array of cells that have accumulated diverse genetic, transcriptional, and post-transcriptional changes during disease progression^1,2^. As such, the potential of these cells to contribute to disease progression or treatment response may vary widely throughout the tumor.

Patient-derived xenograft (PDX) models, whereby surgically resected tumor fragments are directly implanted in immuno-deficient mice and passaged *in vivo*, are increasingly becoming key, renewable, resources for research on solid tumors. Studies have shown that PDX tumors faithfully retain bulk properties of the tumor, such as basic tumor architecture, genetic aberrations and drug sensitivity of the originating tumor^3–8^. However, few resources and tools are available to assess the degree to which PDXs are reliable models of heterogeneity^9,10^. Moreover, the spatial distribution of cellular heterogeneity within PDX tumors is poorly understood. Thus, there is a need to develop approaches for comparisons across PDX models.

**Fig. 1:**
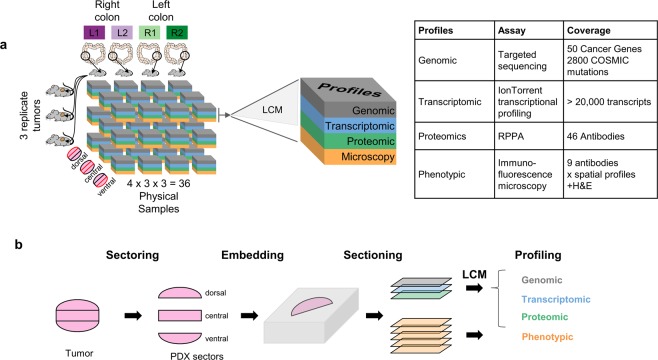
PDX resource for investigating phenotypic and molecular heterogeneity across different length scales and readout modalities. (**a)** Establishment of dataset. Four PDX models (2 from the right and 2 from the left colon) were expanded in 3 replicate tumors, each of which was divided into 3 sectors (dorsal, central and ventral), providing a total of 4 × 3 × 3 = 36 PDX tumor samples. Each sample was profiled using 4 different assays: (i) genomics (targeted sequencing), (ii) whole transcriptome RNA profiling, (iii) proteomic profiling using RPPA, and (iv) microscopy (bright-field H&E staining and immunofluorescence microscopy); samples for assays (i–iii) were processed first by Laser Capture Microscopy (LCM). (**b)** Sample preparation. Each PDX tumor is divided into dorsal, central and ventral sectors, each of which was snap frozen and embedded in OCT; blocks were sectioned; and sections were used directly for microscopy imaging or subject to LCM to isolate tumor lysates for DNA, RNA or RPPA profiling.

Here, we developed a large-scale data resource^11^ to investigate topographic properties of heterogeneity within and across PDX tumors, as well as to spark tool development in this area of research. We chose to focus on metastatic colorectal cancer (mCRC), for which PDXs are well established as a faithful and useful model system^10,12–15^. CRCs exhibit well-characterized primary tumor location (left vs. right colon) and molecular subtype^16^ heterogeneity. Certain histological variants of CRC show a higher degree of tumor heterogeneity than others, with microsatellite unstable (MSI) CRC in particular showing high levels of morphologic heterogeneity^17^. For our study, we selected four models of mCRC, two originating from the right colon (PDX-R1, PDX-R2) and two originating from the left (PDX-L1, PDX-L2) colon (see Table 1 for additional information on the PDX models). We performed genomic, transcriptional, proteomic and H&E and immunofluorescent (IF) microscopy assays on 36 PDX tumor regions, derived from 3 anatomical sectors (ventral, central and dorsal subcutaneous orientations) each taken from 3 replicate tumors from the four PDX models of mCRC (Fig. 1a, Methods). In addition, our dataset includes: growth curves for all harvested tumors, pathologist’s annotation of each tumor region, a mapping of all sections, regions, tumors and models, quality control data for transcript profiling and Reverse Phase Protein Array (RPPA), pure mouse (DNA, Methods) and mouse spike-in data (RNA, Methods) to test for residual effect of mouse cells, and pure mouse tissue stained and imaged for our panel of IF probes. These data are available at https://nciphub.org/resources/2205^11^.

**Table 1:** Clinico-pathologic annotation of PDX models. 5-FU: 5-fluorouracil; Ox: oxaliplatin; Ir: irinotecan; Cet: cetuximab; Bev: bevacizumab.

Model	PDX-L1 (footnote 1)	PDX-L2	PDX-R1^*^	PDX-R2 (footnote 1)
Primary tumor location	Left colon	Left colon	Right colon	Right colon
Biopsy site	Vaginal wall	Ovary	Liver	Periumbilical soft tissue
Patient sex	Female	Female	Male	Female
Age at diagnosis	64	61	28	62
Race/ethnicity	White/non-hispanic	White/non-hispanic	White/non-hispanic	White/non-hispanic
Histology^†^	Mucinous Adenocarcinoma, *pools of mucin*, *segmental and dirty necrosis*	Adenocarcinoma, not otherwise specified	Adenocarcinoma, *extensive angiolymphatic invasion*	Adenocarcinoma, not otherwise specified
Stage at diagnosis	T3N1M0	T3N2M1	T4N2M1	T3N0M1
Tumor grade	Intermediate	Low	High	Unknown
Genetics^‡^	BRAF (V600E)	TP53 (c.96 + 1 G > T)	BRAF (V600E), TP53 (I195N), PIK3CA (G1049S)	BRAF (V600E), TP53 (R175H)
Lines of chemo prior to biopsy	3	1	3	2
Prior chemotherapy agents	5-FU, Ox, Ir, Cetux, Bev	5-FU, Ox	5-FU, Ox, Ir, Cetux, Bev	5-FU, Ox, Ir, Bev
PDX passage #	6	1	2	5

*Genetic and proteomic profiling also performed on patient tumor biopsy sample; response to targeted therapies was recapitulated in the xenograft model^15^.

**Fig. 2:**
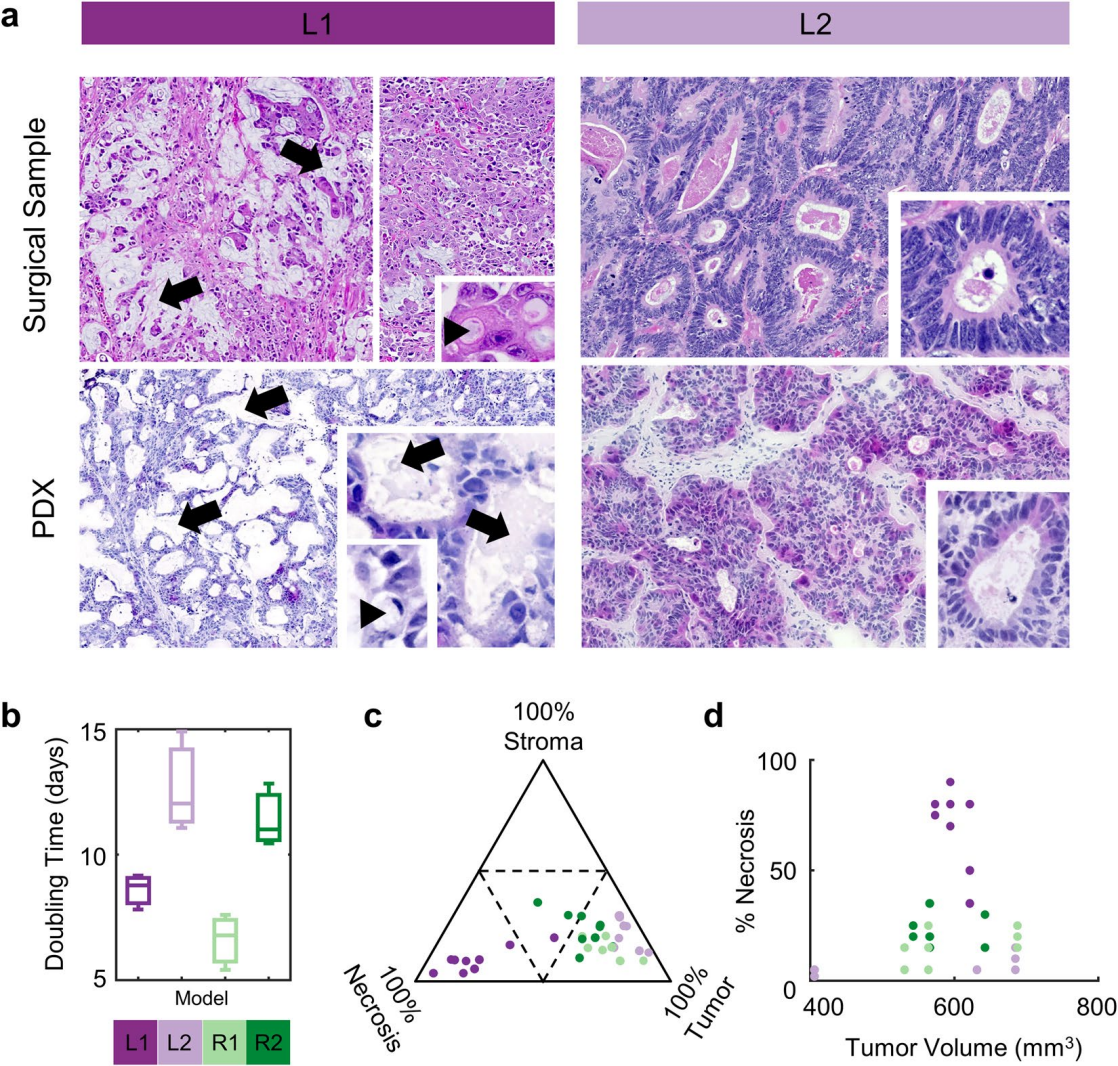
Growth and histological profiling of the PDX model. (**a**) Histopathologic Features of Matched Surgical (Top Row) and PDX Specimens (Bottom Row) of Colorectal Adenocarcinomas. For Model L1 (left column), the surgical tissue (top) from a metastatic implant on the vaginal wall has the typical morphologic and cytological features of a mucinous adenocarcinoma with conspicuous lakes of extracellular mucin (arrows) and abundant cells with intracellular mucin accumulation (arrowhead, inset) arranged in small epithelial clusters and sheets of more amorphous epithelial arrangements. A xenograft (bottom left) derived from this surgical tissue shows similar cellular arrangements and large extracellular spaces consistent with extracellular pooling of mucin (arrows). Higher magnification shows both extracellular and intracellular mucin accumulation with conspicuous intracellular vacuolization (arrowhead, inset). For model L2 (right column), the surgical tissue (top) features of a moderately differentiated adenocarcinoma with abundant, irregular tubules and ribbons of epithelial “glandular” structures composed of columnar tumor cells. Note the markedly enlarged and hyperchromatic nuclei with a paucity of mucin production (inset). A xenograft derived from this surgical tissue (bottom right) demonstrates very similar abundant morphology without conspicuous mucin production. Higher magnification shows columnar cells with similarly enlarged and hyperchromatic nuclei in these “glandular” epithelial arrangements. (**b**) Growth rate is model dependent. Tumor volume growth curves were fit to exponential distributions to extract doubling times (n = 3 for each model). (**c**) Histopathology is model dependent. H&E stained sections of tumors were scored by a pathologist (S.V.) to estimate the percentage of tumor, stroma and necrosis, visualized in a ternary plot. (**d**) Tumor volume does not determine percentage of necrosis. In (**c**,**d**): points are a single section from one of the 36 PDX samples, and colors represent PDX models.

^†^Histology of patient tumor was conserved in the corresponding xenograft (Fig. 2a).

^‡^Mutations common to patient tumor and xenograft model; all cancers were microsatellite stable.

We had three goals in developing this PDX resource for the community. First, our study provides experimental guidelines and best practices to investigate PDX tumor heterogeneity. Central to our design was controlling PDX size to minimize necrotic tissue, preserving the ability to analyze and compare PDX heterogeneity along the ventral/central/dorsal axis of implantation, and evaluating the use of LCM to minimize mouse contributions. A particularly novel feature is that for the genomic, transcriptional and proteomic assays, which perform “bulk” characterization of the PDX regions, laser capture microdissection (LCM) was used to enrich for tumor cells before assaying (Methods). Second, we provide a multi-modal dataset for studying PDX tumor heterogeneity across distance scales. Our large (2TB) data set profiles tumors across genetic, transcriptomic, proteomic, histological and phenotypic axes. To our knowledge, this is one of the largest tissue IF data sets that enables detailed study of single-cell heterogeneity in its spatial context, paired with DNA, mRNA and protein characterization of tumor regions. The large tissue images, across multiple serial sections stained using different readouts (H&E and 8 IF biomarkers, × 3 replicates sections), makes this data set suited for training deep learning classifiers for in-silico microscopy^18^ or 3D reconstruction, though we did not explore these directions in the present work. Third, our study provides approaches and methodology for mapping spatial variation of molecular changes, and for relating these variations to tumor-level properties, such as cancer-type classification and anatomical orientation of PDX tumor.

How microenvironment contributes to tumor heterogeneity, how heterogeneity varies at different length scales within and across tumors, and how best to sample heterogeneity are central questions in PDX studies and cancer biology. Our PDX resource contains all SOPs, metadata, and data—from raw to fully processed—as well as links to software used in our study. We note that the extensive amount of data generated per PDX necessitated a tradeoff in the number and types (Table 1) of PDX models included in this study and does not provide a comprehensive survey of CRC as a whole. Nevertheless, these resources enable the community to extend the datasets and analytical tools to other PDX models, as well as to apply these methodologies to primary human cancer tumors.

## Methods

### Study approval

Biopsies were obtained following patient consent and approval by the UCSF Institutional Review Board (# 12-09139 and 13-2574). This study and all animal work and care were approved by the IACUC at University of California, San Francisco.

## Experimental Methods

### Xenograft

#### Obtaining human samples

A pathologist at gross path review resected a ~1 cm × 1 cm portion (or less) of non-necrotic tumor (so as to not interfere with the interpretations of margins or nodal status). Tissue that was ready for implantation was placed in RPMI (~10 mL in a 50 mL conical tube) and put on ice; otherwise it was snap frozen and stored at −80 °C.

#### Implantation

Samples that were previously frozen were thawed to room temperature before implantation. Working under sterile conditions, tissue was placed in a petri dish with PBS and divided into 6–8 chunks of tissue with size ~1–2 mm^3^ (these are P zero (P0) or passage indicated on vial). The mice used in this study are NOD scid gamma mice purchased from Jackson Laboratory (Stock# 005557|NSG). Up to 10 mice were anesthetized at a time; their skin was cleaned on their upper flank (midback to shoulder region) with betadine solution, then with isopropanol wipes. Two subcutaneous (sc) pockets, bilateral (2 implants per mouse) were created by cutting a small incision (~5 mm) on the back of the mouse. A piece of tissue was inserted into each sc pocket. Tumors were measured twice per week until the tumor volume reached ~600 mm^3^, then the tumors were harvested.

### PDX extraction

Mice were euthanized by exposure to CO_2_ and confirmed dead by cervical dislocation. Tumors, located in either one or both sc pockets, were extracted so as to cut off as much surrounding murine tissue as possibly without disrupting the tumor. The tumor was cut into three sectors (dorsal, central, ventral), snap-frozen immediately and stored in a vial at −80 °C.

### Embedding and Sectioning of PDX

PDX samples were embedded into optimal cutting temperature (OCT) and stored at −80 °C until ready for sectioning. A total of 40 cryosections were prepared on a cryostat for each sample; they were prepared as alternating 8 μm and 4 μm sections (5 each). The 8 μm sections were mounted on uncharged glass slides and used for the isolation of pure populations of tumor cells via Laser Capture Microdissection (LCM). The 4 μm sections were mounted on charged microscope glass slides (FisherBrand, Cat# 12-550-17) and used for immunofluorescent (IF) staining. Slides were stored at −80 °C until further processed. Tumor content for each sample was verified by a certified pathologist before dissection, and the pathologist report is available on the NCI data website.

### LCM

Pure tumor cells were isolated via LCM for downstream DNA, RNA, and protein profiling. Serial sections were dissected for the three molecular analyses to minimize heterogeneity due to changes in the tissue composition across the tumor. Slides dedicated to the DNA/RNA analysis were stained before dissection using the HistoGene LCM Frozen Section Staining Kit (Life Technologies, Carlsbad, CA) following manufacturing instructions. Slides dedicated for the RPPA analysis were stained as previously described (Baldelli, 2015). Isolation of the tumor cells was performed using the Pixcell II IR system (Arcturus Bioscience, Mountain View, CA) and dissected cells were collected on CapSure Macro LCM caps (Arcturus Bioscience, Mountain View, CA). ~2,500 cells were collected on a single CapSure Macro LCM caps for DNA and RNA analyses. LCM for RPPA differed from this procedure in two ways. For every PDX sample, we captured ~4,000 tumor cells as well as residual tissue after removal of tumor regions. For a selected subset of five samples, we additionally captured pure stromal tissue. The above workflow is depicted in Fig. 1b.

### Immunofluorescence (IF) staining

All solutions for IF staining were made in 1X PBS (10X PBS diluted with ddH_2_0, Lonza 17-517Q). All wash steps were performed three times using 1X PBS. Primary and directly conjugated antibodies were purchased from Cell Signaling and stored according to supplied data sheets. Slides were removed from the −80 °C freezer and left at room temperature for several minutes. Tissues were fixed in 4% paraformaldehyde (Electron Microscopy Sciences 15710, Hatfield, PA) for 15 min, washed and permeabilized with Methanol at -20 °C for 10 min and washed once. Tissue was circled with a PapPen and blocked with Universal Blocking Solution (1% BSA, 0.1% Fish skin gelatin, 0.5% TritonX-100, 0.05% Na-Azide in 1X PBS). Slides were incubated overnight at 4 °C with primary antibodies (Table 2): pS6 (MS1, 1:100 dilution, CST 2211), pERK (MS2, 1:50 dilution, CST 4370), pStat3 (MS3, 1:200, CST 9145) and pVEGFR (MS4, 1:50 dilution, CST 2478). Samples were then washed and incubated for 1 h at room temperature with directly conjugated antibodies: β-catenin (MS1, 1:50 dilution, CST 2849), Ki67 (MS2, 1:100 dilution, CST 11882), E-cadherin (MS3, 1:100 dilution, CST 3199), pAKT (MS4, 1:200 dilution, CST 43506). Tissue was then stained with 1:1000-diluted secondary antibodies (goat anti-rabbit Alexa Fluor 568, Life Technologies A11011) and 300 nM DAPI (Life Technologies D1306) and incubated for 1 h and washed again. Tissue was mounted with Prolong Gold Antifade Mountant (Life Technologies P36930) and left to harden for 24 h. The slides were sealed with clear nail polish and stored in the dark at −80 °C.

### H&E

Three slides per tissue sample were stained with H&E. Slides were removed from the −80 °C freezer and left at room temperature for several minutes. They were immersed in 0.1% Mayers Hematoxylin (Sigma MHS-16) for 10 min, washed with ddH2O and then dipped into 0.5% Eosin (Sigma E6003-25G) several times. Slides were then washed with increasing EtOH concentration starting at 50% and ending at 100%. Lastly, the slides were dipped in Xylene before letting them dry, mounting with Permount® Mounting Medium (Fisher Chemical SP15-500) and letting them harden overnight. Finally, slides were sealed with clear nail polished and stored in the dark at room temperature.

### Image acquisition

Immunofluorescence images were acquired using the Leica Aperio Scanscope FL. Per marker set, exposure times were kept constant for the FITC, TexasRed and Cy5 channels. The exposure time for the DAPI channel was set by the microscope, as it uses the DAPI signal to optimize Aperio’s focusing algorithm. We set a focus offset of 0.002 mm. H&E images were acquired using the Aperio Scanscope CS. Tissue detection, focus points and calibration were set by the Aperio Software ScanScope Console.

### Reverse phase protein array (RPPA)

Samples were obtained from either: whole-tissue with no LCM (all 36 samples), ~4000 LCM captured tumor cells (all 36 samples), residual tissue after LCM (all 36 samples), and stromal tissue (5 samples). LCM caps were lysed in a 1:1 solution of 2x Tris-Glycine SDS Sample buffer (Invitrogen Life Technologies, Carlsbad, CA) and Tissue Protein Extraction Reagent (Pierce, Rockford, IL) to which 2.5% 2-βmercaptoethanol (Sigma, St. Louis, MO) was added. Samples were lysed as previously described^19^. Tissue lysates were printed in triplicates onto nitrocellulose coated glass slides (Grace Biolabs, Bend, OR) using an Aushon 2470 arrayer (Aushon BioSystems, Billerica, MA) along with reference standards for internal control. Before immunostaining, each array was treated with Reblot antibody stripping solution (Chemicon, Temecula, CA) for 15 minutes followed by 2 washes in PBS and a one hour block in I-block solution (Tropix, Bedford, MA). Immunostaining was performed at room temperature using an automated Autostainer (Dako Cytomation, Carpinteria, CA).

To reduce background signal generated by endogenous proteins, arrays were first probed with 3% hydrogen peroxide, avidin/biotin blocking system (Dako Cytomation, Carpinteria, CA), and an additional serum free protein block (Dako Cytomation, Carpinteria, CA). Each array was then probed with one primary antibody targeting the protein of interest. A total of 46 (of the 48 probed) antibodies were used for this analysis (list available on the NCI data website). Antibody specificity was tested by western blotting using a panel of cell and/or tissue lysates before being tested on the arrays^20^. Negative control slides were incubated with antibody diluent alone [goat antirabbit IgG heavy + light (1:7,500; Vector Laboratories, Inc. Burlingame, CA) and rabbit antimouse IgG (1:10; Dako Cytomation, Carpinteria, CA)]. A commercially available tyramide-based avidin/biotin amplification kit [Catalyzed Signal Amplification System (CSA; Dako Cytomation Carpinteria, CA)] couples with the IRDye 680RD Streptavidin (LI-COR Biosciences, Lincoln, NE) were employed for the amplification and detection of the signal^19^.

Protein concentration of each sample was quantified on selected arrays using a Sypro Ruby Protein Blot Stain (Molecular Probes, Eugene, OR) protocol following manufacturer’s instructions. Antibody- and Sypro Ruby-stained slides were scanned on a laser PowerScanner (TECAN, Männedorf, Switzerland). Images were analyzed using the MicroVigene software version 5.1 (Vigene Tech, Carlisle, MA), a commercially available software that performs spot finding, background/secondary antibody slide(s) subtraction, and normalization to the amount of protein in each samples determined by the Sypro Ruby Protein Blot staining.

### DNA and RNA extraction

DNA and RNA extraction was performed using the Arcturus PicoPure DNA Extraction Kit (Life Technologies, Carlsbad, CA) and the Arcturus PicoPure RNA Extraction Kit (Life Technologies, Carlsbad, CA) following manufacturing instructions. 8 uL of buffer were added to each cap for the extraction. Samples were incubated at the recommended temperature for 3 hours for the DNA extraction and 30 minutes for the RNA extraction.

### DNA profiling

The Ion AmpliSeq™Cancer Hotspot Panel v2 is a pool of primers used to perform multiplex PCR for preparation of amplicon libraries from genomic “hot spot” regions that are frequently mutated in human cancer genes. DNA libraries were prepared from 10 ng of laser-captured tumor or NIH3T3 DNA samples using the Ion Ampliseq Library Kit 2.0 with the 207 Ion AmpliSeq Cancer Hot Spot panel primer pairs. Individual samples were amplified for a total of 20 cycles of PCR. The amplified product was digested, bar-coded sequencing primers were ligated to the amplicons, and the reactions were cleaned per Ion Torrent procedure instructions. Completed libraries were quantitated by qPCR using primers specific to the A and P1 adaptors. All 37 samples were diluted to 100 pM and combined in equal volumes. The combined libraries were then diluted to 50 pM and loaded into the Ion Chef for automated templating, enrichment and loading onto Ion Torrent PI chips for semiconductor sequencing.

### RNA profiling

The Arcturus PicoPure RNA Isolation Kit was used for RNA extraction on the LCM samples. Each sample was bound to an RNA purification column, DNase treated, washed and eluted in a final volume of 14 µl. The Qubit RNA High Sensitivity Assay was used for quantitation. For samples with a measurable concentration (i.e., more than 5 ng/μl), 1 ng of RNA was used for input into library preparation.

The Ion Ampliseq Transcriptome Kit^TM^ was used for the library preparation and the procedure was performed accurately and according to protocol. Briefly, approximately 1 ng of total RNA was reverse transcribed using the AmpliSeq transcriptome VILO master mix. cDNA synthesis was verified by qPCR on 0.5 µl of the reverse transcription reaction using AmpliSeq specific GAPDH primers. The remaining library input cDNA was amplified for 15 cycles using the AmpliSeq Human Transcriptome Gene Expression kit primer pool with AmpliSeq PCR Master Mix. Amplicons were digested with the proprietary FuPa enzyme, and barcoded IonXpress adapters were ligated onto the target amplicons. The library amplicons were bound to magnetic beads, and residual reaction components were washed off. Libraries were eluted and individually quantitated by qPCR using Ion Torrent P1 and A sequencing primers and SYBR Green master mix. Samples were diluted to 100 pM and combined in equal volumes. The combined libraries were then diluted to 50 pM. Emulsion PCR, templating and PI chip loading was performed with an Ion Chef Instrument. Sequencing was performed on an Ion Proton TM sequencer, with HiQ sequencing chemistry. Analysis was performed using recommended Torrent Suite software with AmpliSeq transcriptome and Ion Torrent Cancer Hotspot panel specific plug-ins.

To test for the potential contribution of residual mouse RNA to AmpliSeq-determined gene expression, an additional sample was generated by adding RNA extracted from mouse NIH3T3 cells to one of the human tumor RNA samples. RNA was combined at a ratio of 10% mouse RNA plus 90% tumor RNA. The mouse spike-in AmpliSeq transcriptome library was prepared as above.

## Analytical Methods

### Tumor growth

Tumor growth was quantified by fitting log of measured tumor volume as a linear function of number of days since implantation (The doubling time was calculated by log(2)/slope) (Fig. 2). To test whether the tumor model explained any of the variation in tumor doubling time, we performed a one-way ANOVA on the doubling time values for our 12 (4 models × 3 replicate PDX) tumors with the 4 model numbers as a grouping variable.

### DNA profiling

The Ion AmpliSeq™Cancer Hotspot Panel v2 is designed to cover approximately 2,800 COSMIC mutations from 50 oncogenes and tumor suppressor genes. Prepared libraries were sequenced on the Ion Torrent Proton instrument. After sequencing, automated Cancer hotspot variant analysis was performed with Torrent Suite v5. Software on the Ion Proton Torrent Server.

#### Quality control

**Fig. 3:**
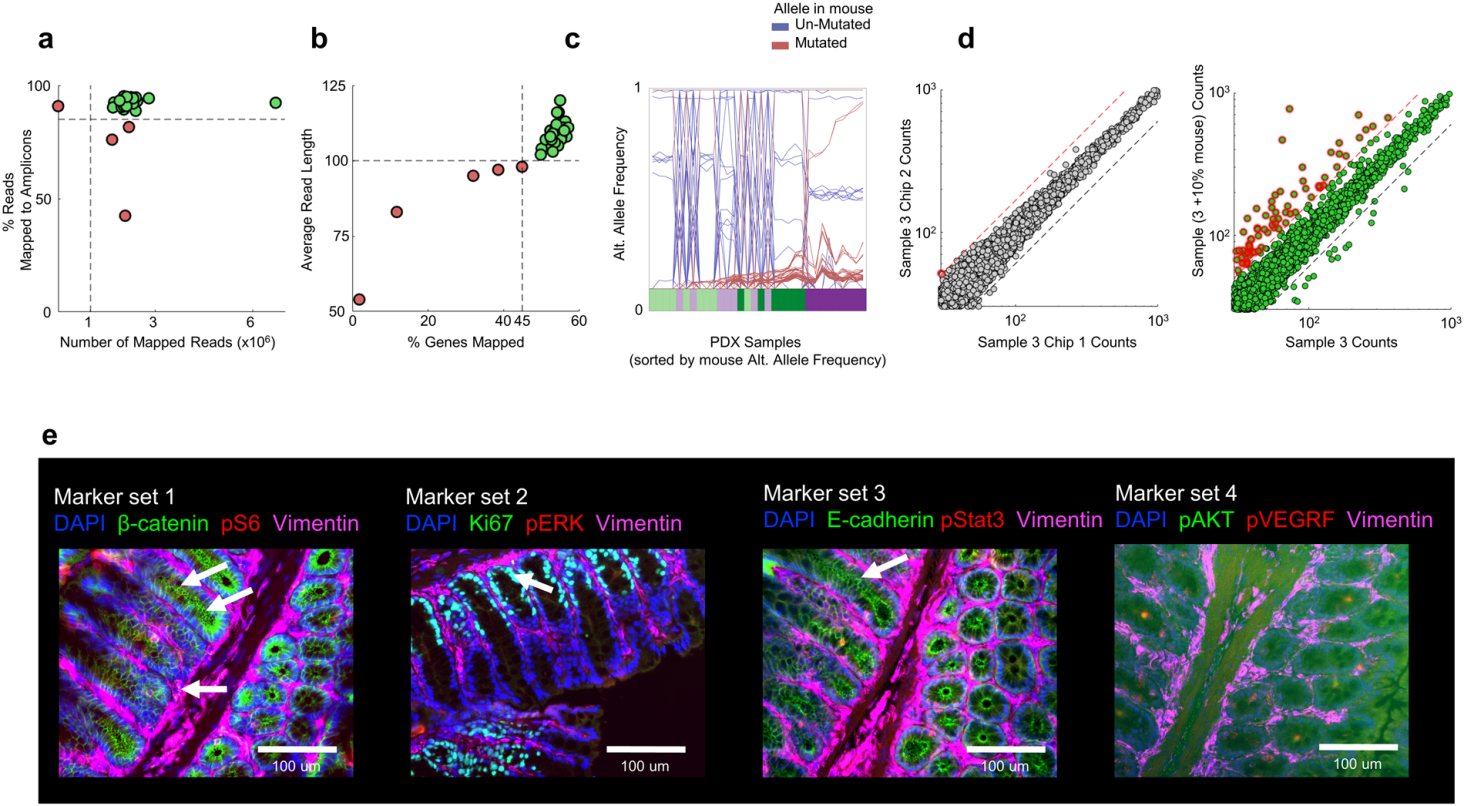
Assay quality. (**a**) DNA Quality Control. Points: PDX tumor sample that did (green) or didn’t (red) meet quality control standards. Sample quality was empirically based on: the total number of mapped reads (set to be >10^6^, vertical dashed line); and the percentage reads mapped to expected amplicons (set to be >85%, horizontal dashed line). (**b**) RNA Quality Control. Points/colors as in a. Sample quality was empirically based on: the % of ~22 K genes that had reads mapped to them (set to be >45%, vertical dashed line); and the average length of reads (set to be >100, horizontal dashed line). (**c**) DNA Mouse contribution. DNA sequencing was run on PDX and pure mouse samples; mutation calls were made against the reference human genome. Plot depicts variant Allele Frequency (y-axis) in our PDX samples as a function of PDX sample (x-axis) for loci (curves) that are called as mutated in mouse (red) or not (blue). The PDX samples are sorted (x-axis) based on the variant allele frequency across the loci that are mutated in mouse (i.e. the red curves go up from left to right). Color bar: PDX model. Note: Loci that are called mutant in mouse and human are candidate false positives, i.e. potentially unmutated in human and driven purely by mouse contribution. The fraction of mutant reads would be expected to co-vary across loci depending on the degree of mouse contamination. (**d**) Effect of mouse on RNA. Scatter plots comparing expression of “non-low” expressing genes for sample #3 either: left) from a pair of technical replicates in the main experiment, or right) with (y-axis) and without (x-axis) a spike-in of 10% mouse. Genes are colored by whether they do (red) or do not (green) lie above the red dashed line (2*(x - y)/(x + y) > 0.5). (**e**) Sample IF images of marker sets in healthy mouse colon. Arrows indicate expected staining patterns, e.g. crypt localization for Ki67 showing proliferating cells (MS2), localization of β-catenin and E-cadherin at cell-cell junctions (MS1 and MS3), and extracellular staining by Vimentin (all MSs).

We used the following quality control measures to ensure the reliability of our profiles: (1) # mapped reads: low coverage implied poor profiling of the genome; and (2) % reads mapped to the amplicon: a low fraction suggested that a samples’ DNA was damaged in some way. Our heuristic criterion for sample inclusion was: # mapped reads >1E6, and % reads mapped to the amplicon >85%. 4 of the 36 samples did not meet these criteria (Fig. 3a) and were not used in further downstream analyses.

#### Mouse contribution

We also performed DNA sequence on pure mouse tissue, using the same pipeline as for the PDX samples, including alignment to the human reference genome. We found that several sites with variant calls made on our PDX samples were also called as “mutant” in the pure mouse. The variant allele frequencies at most of these sites varied in synchrony across the various samples (Fig. 3c). This co-variation is consistent with calls driven by residual mouse DNA, with variation across samples arising from different amounts of mouse DNA in each sample. Accordingly, for all further analysis, sites with pure mouse mutation calls were not used for downstream analysis.

### RNA profiling

The AmpliSeq human transcriptome gene expression primer pool specifically targets 18,574 protein-coding mRNAs and 2,228 non-coding ncRNAs based on UCSC hg19. Ion Torrent AmpliSeq data were analyzed using the Ion Torrent Mapping Alignment Program (TMAP), included with Torrent Suite Software v5. Briefly, by mapping reads onto the known amplicon sequences, the software quantifies the expression of a gene in terms of the number of reads from each specific amplicon. RNA expression levels may be displayed as counts, or as reads per million (RPM), normalized for the number of sequence reads per sample.

#### Quality control

We used the following quality control measures to ensure the reliability of our profiles: (1) average read length: based on the size of our amplicons, the average length of mapped reads is expected to be ~100 base pairs. Shorter read lengths were suggestive of poor quality reads; and (2) % genes mapped: this is the fraction of the full set of 22 K genes that has some reads mapped to it. Given our total RNA levels, we expected to be able to map ~50% of the genome, with lower values indicating poor quality data. Our criterion for sample inclusion was that the average read length be greater than 100 and 45% of the genes be mapped. As with the DNA, these cutoffs were visually selected to exclude clear outliers. 5 out of 36 samples did not meet these criteria (see Fig. 3b) and were not used in further downstream analysis.

#### Normalization

For each sample in the main experiment (but not the mouse spike-in), RNA sequencing was performed on 4 replicate chips. The number of reads were Reads-Per-Million (RPM) normalized, i.e. the total number of reads for each sample across as all genes was equal to 1 million. The reported RNA expression per sample was the average of the RPM values from the four replicate chips.

### Pathway scores

For each of our samples deemed to have good-quality RNA, their RNA expression profiles were used to calculate relative up or down regulation of the 50 pathways (Hallmark gene set from MsigDB^21^) using GSVA^22^. Briefly, for each gene, GSVA first calculates the extent to which the gene’s levels are higher/lower than in a specific sample relative to the entire collection of samples. Then for each gene set (i.e. Hallmark Pathway), GSVA scores a sample based on the extent to which there is an over representation of high/low expressing genes for that sample.

### Immunofluorescence microscopy

#### Image correction

The IF images were observed to display two types of imaging artifacts: a) a non-periodic but slowly varying intensity offset that caused the intensity in non-tissue parts of the image to be non-zero, and b) a striping pattern that modulated image intensities periodically with an image/marker specific shading pattern but fixed periodicity of 1560 pixels along the x-axis. Thus, we assumed a model of *I*_*obs*_ = (*I*_*true*_ + *B*)*S*, where *I*_*obs*_ and *I*_*true*_ are the observed and “true” images, *B* is the background intensity, and *S* is the periodic shading pattern. We estimated these corrections from non-tissue portions of the image (where, in principle, *I*_*true*_ = 0) on a per-channel and image basis and applied corrections to the tissue intensities. *B* was estimated as follows: we divided the image into large blocks of height 480 pixels and width equal to the striping pattern, calculated average intensities in blocks that contained no tissue, and used a Mumford-Shah infilling approach^23^ to estimate *B* within the tissue. *S* was estimated as follows: the correction at a given phase of the periodically varying vertical stripes was obtained by averaging *I*_*obs*_/*B* non-tissue pixels each period. “True” image intensities were obtained from the model above as ${I}_{true}=\frac{{I}_{obs}}{S}-B$.

#### Intensity normalization

To reduce the contribution of non-biological variations on observed biomarker intensity differences between samples, we performed intensity normalization as follows. To avoid overfitting, we use linear normalization (i.e. we transformed the intensities of all pixels in a biomarker image using the same linear function). We performed two marker-specific normalizations. First, for DAPI, we sought to reduce the effect of exposure time variation on image intensity. In our imaging experiments, DAPI was the only biomarker for which the same exposure time was not used across all samples. We found that DAPI intensities varied linearly with exposure time, and we therefore simply scaled DAPI intensities such that each image received an effective exposure time of 125 ms. Second, for each biomarker, we sought to reduce the impact of technical slide-to-slide variation on observed intensities as we are primarily interested in the biological variation between samples. For each of our 36 samples, the 3 replicate sections stained with the same markers should exhibit similar intensity profiles, assuming no slide-to-slide intensity variation. Therefore, we linearly transformed the intensity on each section such that the 25th and 75th percentiles across these replicate sections were equal^24^. The numerical value of the 25th and 75th percentiles was chosen as the average of the 25th and 75th percentiles across the replicate slides.

### Marker tumor intensity

**Fig. 4:**
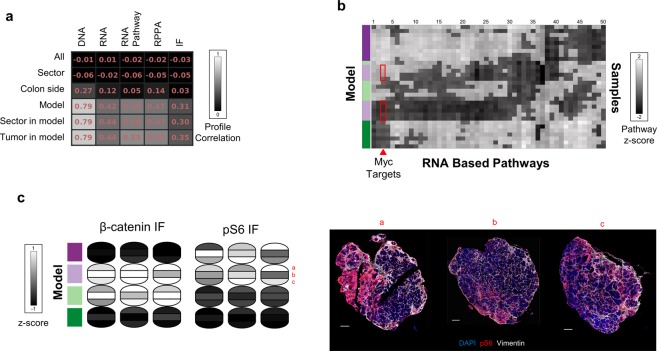
Multi-assay variation of heterogeneity. (**a)** Analysis of PDX tumor heterogeneity. Correlation of PDX samples profiles across different scales (rows) and measurement modalities (columns). For every distance scale (e.g. model) we report the average pairwise correlation across all pairs of samples at that scale (e.g. any two samples from the same model). (**b)** Pathway transcriptional expression heterogeneity within and across tumor models. Grayscale: upregulation of a hallmark pathway (column) in a sample (rows). Rows and columns were sorted using unsupervised hierarchical clustering. Red squares: intra-tumoral heterogeneity for case of c-Myc target pathways. (**c)** Left: sector-averaged β-catenin and pS6 levels based on IF expression. Right: tumor images with IF staining from 3 sectors in PDX-L2. Purple/green color bars indicate PDX model.

We devised an automated process to calculate the intensity of markers in tumor (i.e. non-stromal) cells using a combination of standard image analysis approaches and DNA- and stromal-specific markers (Fig. 4c). First, to identify cellular portions of an image, we applied adaptive thresholding to the DAPI channel, which essentially identifies nuclear pixels, and then we performed an image closing operation to fill in the area between nuclei. To identify stromal portions, a similar operation was applied to vimentin, a stromal-specific marker. We then defined tumor regions to be the cellular regions of the image that did not belong to the stromal areas. Finally, mean marker intensity (Fig. 4c) was calculated by averaging over all pixels in the tumor areas of an image.

### Image heterogeneity profiling

To profile the heterogeneity of our IF images, we made use of PhenoRipper, an un-supervised image analysis approach^25^. Given a collection of images, PhenoRipper divides each image into a collection of sub-cellular blocks and identifies a set of stereotypical “block-types” based on the biomarker intensity colocalization patterns exhibited by the pixels in a block. The heterogeneity of an image can then be described in terms of its PhenoRipper profile—the relative frequency of appearance of the different block type in that image—which may be thought of as a description of the mixture of sub-cellular phenotypes. We set the following parameters for PhenoRipper: (1) marker intensity scaling was chosen to be the same for all markers (0 and 35,000 as minimum and maximum, respectively); (2) a block-size of 20 pixels; and (3) an intensity threshold of 20% of the max intensity to distinguish between foreground and background regions. Default values were chosen for all other parameters, including 30 for the number of block types, which led to a 30-dimensional profile vector for each image.

In profiling tissue heterogeneity, we wanted to not just compare heterogeneity across tissues, but also within a tissue. We therefore divided each tumor tissue image into a grid of 1300 × 1300 pixels sub-images and generated PhenoRipper profiles for each of these sub-images. Given our imaging parameters, 1300 pixels roughly corresponds to the size of a 0.6 mm diameter tissue microarray core. PhenoRipper profiles produced a data cube of size *n*_*x*_ × *n*_*y*_ × 30, where a tumor image contained *n*_*x*_ × *n*_*y*_ sub-images and 30 is the length of a PhenoRipper profile for a single image. For applications where we were only interested in the overall heterogeneity of a sample (and not its distribution within the sample), we performed a weighted average of the PhenoRipper profiles across the *n*_*x*_ × *n*_*y*_ sub-images, with each sub-image weighted in proportion to the amount of tissue (i.e. number of foreground blocks) it contained.

### Sample-To-Sample correlation

The expression profiles (genetic/rna/pathway/rppa/if) as described above were z-score normalized for each readout (e.g. gene/pathway/antibody) (Fig. 4a). Readouts with no variation across the full set of samples were not used in correlation calculations. Correlations used in Fig. 4a were calculated based on pairwise Pearson correlations between these normalized profiles.

### Deconvolution of IF marker intensity variance across length scales

For any biomarker, every pixel in an IF stained image can be thought of as belonging to a hierarchical set of levels, spanning length scales from its local sub-cellular neighborhood to the PDX model from which that tumor was derived. Specifically, within an image, we can consider the pixel belonging to growing sets of pixel neighborhoods (with order-of-magnitude length scales): sub-cellular (<10 micron) ⊂ cellular (between 10 to 100 micron) ⊂ micro-environmental (100 to 1000 micron) ⊂ regional (1000 micron to mm scales of slide). Across images, each image represents one of multiple sections from a sector, which in turn is derived from one of three tumors representing one of 4 models. We sought to break down the observed pixel intensity variation (for a biomarker) across the entire collection of pixels across all models, into contributions arising from each of these scales.

Accordingly, we started from the highest scale (whole data), and subtracted out the average intensity across all pixels at this scale (mean intensity of the biomarker). We moved on to the next scale (PDX model), and for each group (model) at this scale calculated the average of the residual intensity. These difference from the group average at this scale were then passed on to the next scale, where the procedure was carried out recursively at increasingly finer levels of grouping until, at the final cellular level, the residuals were considered to represent sub-cellular variation. For the levels above image (i.e. section images ⊂ sector ⊂ tumor ⊂ model ⊂ dataset), we performed a simple non-weighted mean. For levels within an image (image ⊃ region ⊃ microenvironment ⊃ cellular ⊃ subcellular), we performed a weighted average that takes into account the distance between pixels, in a scale-space-theory inspired approach. Specifically, we performed averaging by convolving with Gaussian filters of different widths, σ = *Inf*, 1000,100,10 pixels respectively (1 pixel = 0.4619 microns). We restricted ourselves to nuclear pixels identified as described above. Taken together, the intensity for every nuclear pixel is expressed as a sum of intensities across these different length scales:

$${I}_{p}=\sum _{scale}{I}_{p,scale}$$

where *I*_*p*_ is intensity of pixel p, and *I*_*p*,*scale*_ is the contribution from each specific scale. We defined total variation as *var*_*p*_(*I*_*p*_) and variation explained at each scale as *var*_*p*_(*I*_*p*,*scale*_).

### Heterogeneity sampling

**Fig. 5:**
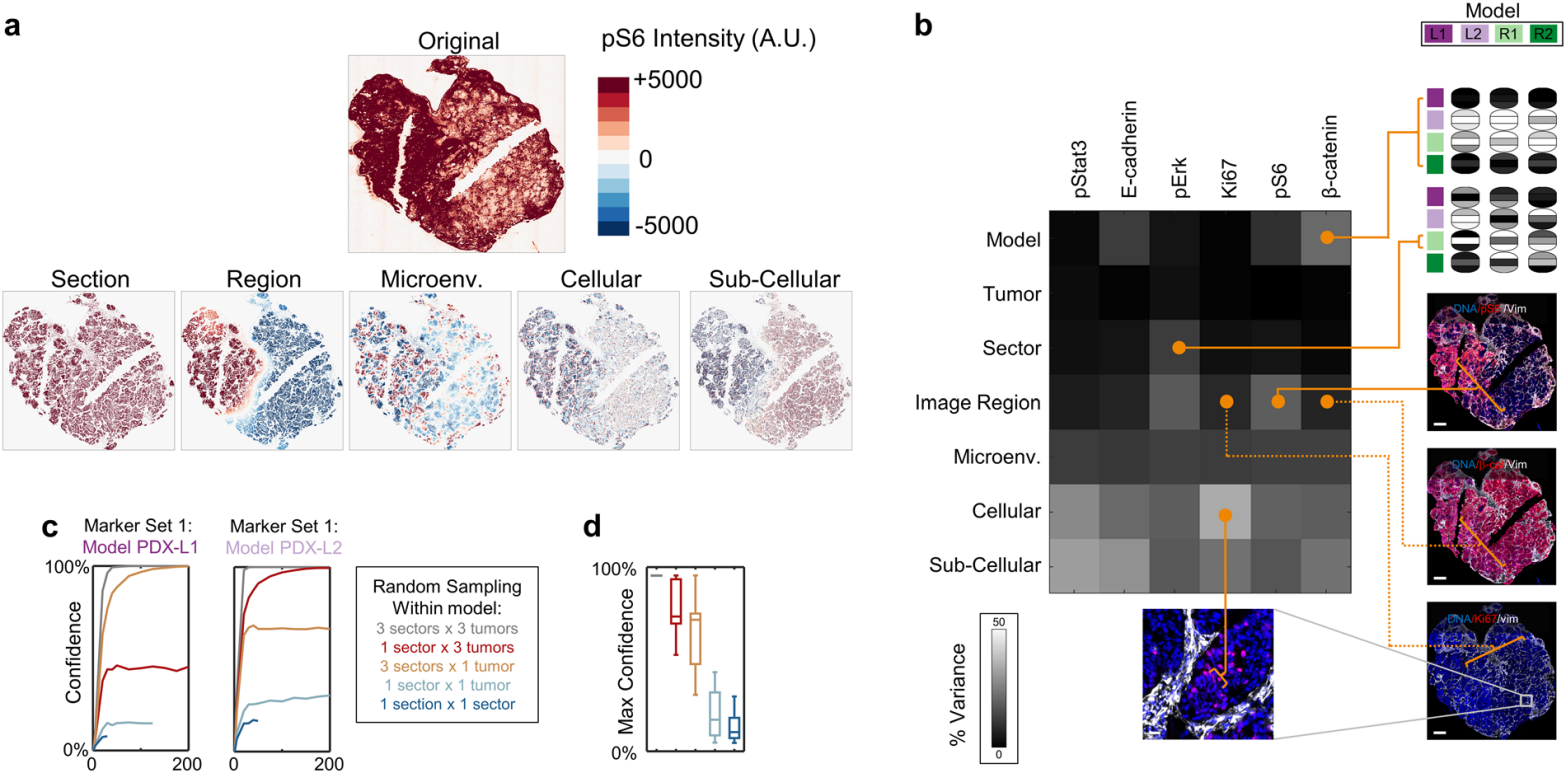
Analysis of intra-sample heterogeneity using IF. (**a)** Multi-scale deconvolution of nuclear pixel intensity variation. Top: pS6 intensity for a tumor section. Bottom: breakdown of the original pattern as a sum of expression at different length scales, from whole section to subcellular. (**b)** Variation for individual biomarkers (columns) across different spatial scales (rows). Grayscale color: percentage of variance in biomarker pixel intensity captured across length scales (columns). Sample images are shown for high vs low (solid vs. dashed orange line) variation at different length scales. (**c)** Confidence that sub-samples from a PDX model capture full model heterogeneity depends on the sampling strategy. Panels: results for images from models PDX-L1 and PDX-L2 respectively, stained for IF marker set 1. Results are averaged over 40 PhenoRipper models. Y-axis: confidence that PhenoRipper profiles of a collection of sub-images “matches” the full model profile. X-axis: number of sub-images in collection. Curves: different strategies of selecting sub-images within a model. (**d)** Box-Whisker plot for the saturation (i.e., highest) confidence level for each sampling strategy across all combinations of models and marker sets.

We wanted to test how many samples were required to capture intra- and inter-tumor variation for a PDX model (Fig. 5c,d). We made use of a recently developed experimental design approach^26^, which requires the user to supply the following elements:

1. a description of the true heterogeneity of each PDX model: here, we used a PhenoRipper profile obtained by combining profiles from all images (3 replicate PDX’s × 3 sectors × 3 sections) for each marker set;
2. a description of the heterogeneity of an arbitrary sub-sample: here, we described heterogeneity in terms of the weighted average of sub-image PhenoRipper profiles as described above;
3. a criterion to determine when a sub-sample accurately captures the heterogeneity of the model: “good” sampling of heterogeneity was defined to be when the average deviation (across the 30 block types) between the “true” and sub-sample PhenoRipper profiles was <0.01;
4. a sampling strategy (i.e. the process of selecting a set of subsamples): here we considered the following sampling strategies for a sampling run with *n* subimages:

- within model: n sub-images selected randomly from all sub-images within a model;
- within tumor: one of the 3 (replicate) single tumors belonging to a model was randomly selected, and *n* sub-images were then randomly selected from this tumor;
- within sector: for each sampling run, one of the three sectors (dorsal/ventral/central) was chosen at random, and then *n* sub-images were selected from this sector, but could come from different tumors;
- within sample: one of the 9 samples per model was chosen at random, and then *n* sub-images were selected from that sample;
- within section: first one of the 27 sections (9 samples × 3 replicates sections per sample) per model was chosen at random and then *n* sub-images were selected from that section.

Given these elements, the method computed the confidence for a given sampling strategy and number of samples. Confidence was defined as the fraction of sampling runs for which the subsamples captured the heterogeneity of the model, as defined above. In our case, we sub-sample 1000 times and calculated the fraction of cases when good sampling (as defined above) was achieved. We averaged our confidence values over 40 PhenoRipper runs.

## Data Records

All data including raw and processed for DNA/RNA/RPPA, H&E and IF stained image data and a description of their organization can be found at^11^:

10.17917/yr13-py25

Additionally, all DNA and RNA sequencing data has been deposited at DDBJ^27^ as a BioProject with accession number PRJDB7951 and study accession number DRP004856^28^.

## Technical Validation

Here, we include analysis intended to help guide users through these datasets. The analyses include: (a) quality control, (b) a multi-modal analysis of heterogeneity across different spatial scales, and (c) a demonstration of how image data might guide experimental design.

### Quality control

We first investigated histopathological properties of our PDX collection. H&E slides were examined by a pathologist (S.V.) who found them histologically consistent with colon adenocarcinoma, displaying no evidence for transition to a different cancer type. Moreover, where we had access to H&E tissue from the parent surgical sample, we found that the characteristic histopathologic features were also captured by the corresponding PDX samples (Fig. 2a). Further, for each of the 36 PDX regions, an H&E section was scored for the percentage of tumor necrosis and mouse stroma and the degree of dysplasia. Consistent with previous findings^10,14^, we found that properties of histology and tissue composition (% of tumor, stroma and necrosis) are largely driven by PDX model (Methods, Fig. 2). Further, we found no dependence of necrosis on tumor volume within our dataset. Taken together, these results suggested that histo-pathology is largely reproducible at intra-tumoral scales within a PDX model.

We performed various quality controls to ensure reliability of our assays. To test whether the process of DNA/RNA extraction from post-LCM lysates negatively impacted these assays, we used the number of reads and extent of mappability as proxies. For both assays, ~90% of samples showed similar high levels of quality; a few outlier samples failed these tests and were excluded from further analyses (Methods, Fig. 3a,b red points). Additionally, we used pure mouse samples as a reference to test the effect of mouse contamination on our profiles. For DNA, we found that despite performing LCM, several mutations called in our samples were present in both pure mouse and in the tumor samples, with sample- and model-dependent distributions of variant calls (Fig. 3c). These calls are likely driven by trace mouse DNA that is included despite the LCM process, and these genes were dropped from downstream analyses. For the RNA, an additional spike-in of 10% mouse RNA did not affect the overall transcriptional profile (Fig. 3d); however, ~100 profiled genes (<0.5% of all genes) showed a large increase in expression in the spike-in relative to the PDX sample (Fig. 3d, points with red outlines). Our analyses suggest that LCM alone is not sufficient to completely exclude mouse contribution from profiling assays, although this effect likely only impacts a tiny fraction of genes.

Quality control of the RPPA datasets was performed at the Center for Applied Proteomics and Molecular Medicine at George Mason University using antibodies validated as described in Methods^20^. Raw images of the protein array slides are included in our data set. For the IF datasets, antibodies were obtained from commercial vendors (Table 2) and staining protocols were optimized on PDX tissue and tested on healthy mouse colon (Fig. 3e). Staining pattern and localization were examined visually for consistency with reported literature. Based on this quality control, all marker panels except MS4 (which showed a diffuse staining pattern) were used in subsequent analysis.

**Table 2:** List of IF Antibodies used in immunofluorescence assays. All antibodies have been tested and verified for human (Hm) and mouse (Ms) reactivity. Marker set 4 (pVEGFR, pAKT) was not used in analysis due to diffuse staining pattern.

Marker set	Biomarkers	Pathway processes	Prod#	Lot	Vendor	Species Reactivity
1	β-catenin	Wnt signaling	2849	8	Cell Signaling Technology	Hm, Ms*
	pS6	Proliferation	2211	23	Cell Signaling Technology	Hm, Ms
2	pERK	MAPK signaling	4370	17	Cell Signaling Technology	Hm, Ms
	Ki67	Proliferation	11882	5	Cell Signaling Technology	Hm, Ms
3	pStat3	JAK/Stat signaling	9145	31	Cell Signaling Technology	Hm, Ms
	E-Cadherin	EMT	3199	15	Cell Signaling Technology	Hm, Ms
4	pVEGFR	Angiogenesis	2478	15	Cell Signaling Technology	Hm, Ms
	pAKT	AKT/mTor Signaling	43506	1	Cell Signaling Technology	Hm, Ms
1/2/3/4	Vimentin	Stromal marker	9856	11	Cell Signaling Technology	Hm, Ms

*Predicted due to 100% sequence homology.

### Multi-modal analysis of spatial heterogeneity

We next investigated how consistent our different molecular profiles were across models, replicate tumors, and sectors. The best correlations (Methods) were seen at the level of the PDX model for all assays, with genetic assays showing the highest levels of within-model concordance (Fig. 4a). Interestingly, across all assays, we did not see an appreciable increase in correlation at higher resolution within the same model (Fig. 4a: rows 4 vs. 5 and 6) or by restricting our analysis to samples that arose from the same anatomical sector (Fig. 4a: rows 1 vs. 2, or 4 vs. 5). These results suggest that within a PDX model, the length-scale of sector had little effect on tumor biology or heterogeneity, an observation that had not been previously tested to the best of our knowledge. Specifically, in our data set, samples from the same sector of replicate tumors were no more similar to each other than samples from different sectors within the same PDX tumor. Overall, these global analyses, across different assay modalities, suggest that the PDX model, more than replicate or region, dominates global molecular characterization.

While the global molecular state is consistent for samples coming from the same PDX model, we did observe intra-model heterogeneity at the pathway-level (Fig. 4b). For example, samples from model PDX-L2 showed heterogeneity of Myc Targets (Fig. 4b, red triangle and rectangles). This intra-model heterogeneity was also observed for pS6 in IF (Fig. 4c). Interestingly, an examination of the IF images for PDX-L2 revealed that the increased pS6 levels did not represent a uniform increase in pS6 across these sectors, but rather the increased occurrence of a pS6-high subpopulation in specific parts of a tumor.

Microscopy-based assays capture phenotypic heterogeneity across length scales, spanning from sub-cellular variation all the way to global differences between PDX models. Motivated by the observed intra-tumor heterogeneity of pS6, we sought to quantify local tumor heterogeneity and examine its spatial variation across these different length scales. Accordingly, we developed a scale-space-theory-inspired method^29^ (Methods, Fig. 5a) to deconvolve the overall observed variation in pixel intensity of IF biomarkers (Fig. 5b, x-axis) into components arising from differences between (Fig. 5b, y-axis): PDX models, replicate tumors within a model, sectors within a tumor, image regions within tumor sections, local microenvironmental areas within regions, cellular variation within the micro-environment and the residual sub-cellular variation.

Our approach revealed distinct spatial patterns of biomarker expression (Fig. 5b). Consistent with results above (Fig. 4c), pS6 and β-catenin showed different expression levels across PDX models, yet their patterns of variation within a model are very distinct: when β-catenin is expressed it appears to be expressed uniformly across the model, whereas there is a pS6 high sub-population that shows regional variation across different regions of a tumor. pErk also shows large variation between image regions in a sector and additionally between different sectors within a tumor. On the opposite end of the spectrum is Ki67, which also shows significant cell-to-cell heterogeneity, but whose high sub-population (representing proliferating cells) seems to be more uniformly distributed across regions, and tumors. Interestingly, there is very little apparent variation between replicate tumors within a model suggesting that, at least for our data, individual tumors are representative of their parent models.

### A demonstration of how image data might guide experimental design

To understand the implications of these patterns of variation on the design of experiments to capture heterogeneity, we synthetically divided each tumor slide image into ~100 sub-images, each approximately the size of a 0.6 mm diameter tissue-microarray core (size of scale bar in Fig. 4c). We then quantified the heterogeneity of each sub-image using PhenoRipper, an un-supervised image profiling approach^25^. Given a set of images, PhenoRipper automatically identifies a set of stereotypical sub-cellular states, and describes the heterogeneity of each image in terms of the frequency of occurrence of these states (Methods)^25^.

Due to spatial heterogeneity present within tumors, profiling only a few small local areas may poorly represent the overall heterogeneity of a PDX model. A rational approach is to sub-sample the PDX model in a way that balances the effort to acquire more tissue with the improved confidence in capturing heterogeneity. Here, we quantified the confidence that the phenotypic profile of a collection of sub-images matches the profile of the full model^26^. The level of confidence increases with the number of sub-samples (Fig. 5c), but our results show that the exact behavior depends on the way the sub-sampling is performed relative to the biology of the model. For example, in the case of marker set 1 and PDX-L1 (Fig. 5c, orange and red curves in left and right panels), adding sub-samples from the same tumor but from different sectors (orange curve) increases confidence more than adding multiple sub-samples for the same sector but from different tumors (red curve); however, the opposite is true for PDX-L2.

For any sub-sampling strategy, a point of diminishing returns for adding more samples can be reached. This “saturated confidence level” reflects the maximum heterogeneity than can be captured with a sampling strategy. To obtain a more general understanding of sampling, we averaged the saturated confidence levels across models and marker sets (Fig. 5d). As might be expected, restricting samples to a single tumor sector or section poorly represents overall model heterogeneity. Given the appreciable increase in confidence observed by sampling across multiple sectors within a whole tumor (Fig. 5d, orange vs light blue), our study suggests this is likely a worthwhile investment. A further increase in confidence is possible by sampling the same sector across multiple tumors, although given the modest gains, the additional experimental effort must be more carefully evaluated (Fig. 5d, orange vs red).

## Data Availability

All code used to process data and generate figures in this paper are available from the Altschuler-Wu Lab public github page: https://github.com/AltschulerWu-Lab/MultimodalPDXHeterogeneity.
